# Chronic kidney disease with malignant peripheral nerve sheath tumor of the ureter: a case report

**DOI:** 10.3389/fonc.2024.1368996

**Published:** 2024-05-02

**Authors:** Xinyang Yin, Xiaodong Wang, Zhenlin He, Xiaolong Chen, Qing Wang, Kehua Jiang

**Affiliations:** Department of Urology, Guizhou Provincial People’s Hospital, Guiyang, China

**Keywords:** MPNSTs, malignant tumors, chronic kidney disease, NF1, chemotherapy, case report

## Abstract

Malignant peripheral nerve sheath tumors (MPNSTs) are a complex group of malignant tumors originating from nerve cells or benign peripheral nerve sheath tumors and are commonly found in major plexus/nerve root sites such as the limbs, head, and neck. Malignant peripheral nerve sheath tumors originating in the ureter are extremely rare. Herein, we report the case of a 63-year-old patient with a malignant peripheral nerve sheath tumor of the right ureter who underwent laparoscopic radical resection of the right kidney and ureter. The patient also had stage 5 chronic kidney disease (CKD). Therefore, chemotherapy and radiotherapy were not considered. No tumor recurrence was observed during the follow-up period.

## Introduction

Malignant peripheral nerve sheath tumors (MPNSTs) are complex malignant tumors of neural origin that account for no more than 10% of soft tissue sarcomas (1, 2). They are classified as neurofibromatosis type 1 (NF1) -associated MPNSTs and sporadic MPNSTs. MPNSTs can occur anywhere in the body but is more common at the location of major plexuses/nerve roots, with the most commonly involved sites being the extremities, followed by the trunk, head, and neck (3). It is rarely seen in the urinary system. This report retrospectively analyzes the management of a 63-year-old patient admitted to our hospital with a malignant peripheral nerve sheath tumor of ureteral origin.

## Case description

**Figure 1:**
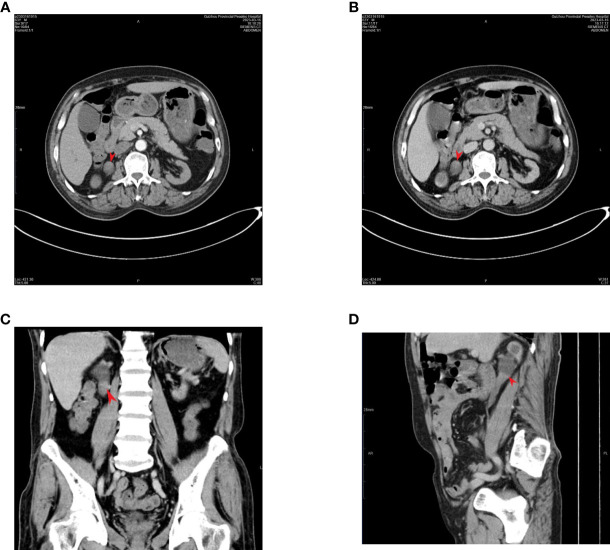
Enhanced CT of the urinary system: a soft tissue mass is visible within the right superior ureter, with mild enhancement noted on an enhanced scan. (tumor location flat against the 2nd-3rd lumbar vertebrae). The arrow points to the tumor: **(A)** arterial phase; **(B)** venous phase; **(C)** coronal plane; **(D)** sagittal plane.

A 63-year-old male patient was admitted to our hospital with a right ureteral tumor. He experienced pain in the right lumbar and back regions, with no signs of urinary tract irritation, hematuria, pyuria, or fever. There was no percussion pain in the renal region or ureteral tenderness, and no intra-abdominal or retroperitoneal masses were initially detected. Enhanced computed tomography (CT) of the urinary system in our hospital (**Figure 1**) showed a nodular soft tissue shadow filling the lumen of the upper section of the right ureter (approximately at the level of the flat lumbar 2-3 vertebrae), with a larger cross-section of approximately 18mm x 17mm, and upper and lower diameters of approximately 36mm. The lesion was mildly enhanced on contrast; the surrounding fat interstices were clear, and there was a slight dilatation of the upper part of the ureter on the right side of the lesion and the pelvic calyxes showed fluid accumulation; the left side did not show any abnormality. There were no obvious abnormal enhancing shadows in the parenchyma of either kidney and no signs of tumors in the bladder, prostate, retroperitoneum, or pelvis. Tumors in the right upper ureter were excluded. No tumors were observed on lung computed tomography. Routine urine examination revealed a strongly positive urinary occult blood test (3+) and a positive qualitative urine protein test. Tumor markers were as follows: carcinoembryonic antigen 9.4 ng/mL and alpha-fetoprotein 1.5 ng/mL. Multiple reviews showed serum creatinine greater than 700 μmol/L and glomerular filtration rate less than 10 ml/min/1.73m^2^; however, the patient denied a history of CKD. The patient was diagnosed with CKD stage 5 and was recommended long-term hemodialysis. The patient was severely anemic with a red blood cell count of 2.26 x 10^^^12/L and hemoglobin of 69.0 g/L; after a total transfusion of 6U of red cells suspensions, he underwent laparoscopic radical resection of the right kidney and ureter under general anesthesia on April 03, 2023. The intraoperative blood loss was approximately 50 ml.

**Figure 2:**
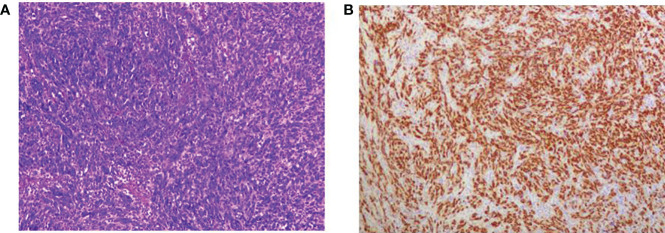
Microscopic view of tumor histopathological sections; **(A)** HE staining x100; **(B)** SOX10 histochemical staining.

Post-operative pathology showed the kidney specimen with part of the ureter and bladder wall measuring 8.5 x 6 x 4 cm, with ureter measuring 21 cm in length and 0.5-1.5 cm in diameter. In the upper part of the ureter, a grayish-white striated mass was seen, measuring 3.6 x 1.8 x 1.6 cm (Classified by the American Joint Committee on Cancer [AJCC] as Class I), solid with medium texture and friable areas, invading the whole layer of the ureteral wall and not involving the renal pelvis, with dilated pelvic calyces and thinning of the renal parenchyma which measured 0.8-1.2 cm in thickness. The rest of the ureter showed no obvious mass. The upper right ureter tumor was a malignant mesenchymal tumor with necrosis, and considered to be MPNSTs measuring 3.6 x 1.8 x 1.6 cm, with vascular invasion, but without neural invasion or tumors elsewhere in the kidney or bladder wall. Chronic pyelonephritis with atrophy of the renal parenchyma, sclerosis of some glomeruli, significant interstitial fibrosis, a large number of lymphoid hyperplasia and lymphoid follicle formations, and unspecified renal hilar vessels was noted (**Figure 2**). Immunohistochemistry (IHC) results were as follows: S100 (+), SOX10 (+), SMA (+), Desmin (localized +), MyoD1 (cytoplasmic +), CD117 (-), CD34 (vascular +), CK20 (-), CK7 (-), panCK (localized +), Dog-1 (-), Melanoma (-), Myogenin (-), P63 (-), STAT-6 (-), and Ki67 (approximately 70%).

The final diagnosis was malignant peripheral nerve sheath tumor of the ureter. The patient’s pelvic drain was removed 3 days after surgery, and the patient was followed up regularly for 8 months and did not receive regular dialysis treatment. There has been no local recurrence or distant metastasis of the tumor to date; however, non-specific clinical manifestations of renal failure such as obvious loss of appetite, poor natriuresis, and fatigue were observed.

## Discussion

MPNSTs are often defined as malignant tumors originating from a peripheral nerve or a preexisting benign nerve sheath tumor (usually neurofibroma) that has deteriorated or a tumor formed by the deterioration of neurofibroma in individuals with NF1 (2). They can occur in any age group, with children accounting for 10–20% of MPNSTs cases (4, 5). They arise from nerve cell components such as Schwann cells, perineuronal cells, or transformation of previously benign peripheral nerve sheath tumors (6); approximately 8–13% of NF1 cases can mutate to progress to MPNSTs, accounting for 50% of MPNSTs; while the remainder of cases are predominantly disseminated, with radiation induction and genetic factors also contributing to the occurrence of MPNSTs (2, 3, 5, 7). Importantly, NF1 is an inherited autosomal dominant disease, involving a tumor suppressor gene on chromosome 17 (2), and the international consensus of the latest revision of the NF1 diagnostic criteria for 2021 suggests that the diagnosis can be made definitively (8). In the current case, the patient’s medical and family histories were examined, and malignancies of NF1 origin were excluded.

Malignant peripheral nerve sheath tumor symptoms are non-specific and imaging is indispensable for tumor screening. Currently, a range of tests, such as CT, magnetic resonance imaging (MRI) and positron emission tomography (PET), are the main tools for evaluating and diagnosing MPNSTs. MRI features such as peritumoral edema, local infiltration with irregularities of margins, and heterogeneous enhancement after tumor contrast appear to be more indicative of MPNSTs (3, 9). Additionally, [18F]2-fluoro-2-deoxy-D-glucose (18F-FDG) PET is a sensitive and specific diagnostic tool for MPNSTs; FDG PET and PET CT identify primary and/or metastatic MPNSTs and provide an estimate of the tumor grade (3, 10–12). Although its sensitivity and specificity vary, FDG-PET (particularly in combination with CT) is a more reliable adjunctive diagnostic tool for MPNSTs and helps determine their prognosis. The diagnosis of MPNSTs requires a combination of clinical presentation, morphology, and IHC owing to a lack of definite immunohistochemical markers (13).

Currently, surgical resection remains the only treatment for limited MPNSTs (3, 6), and negative margins are critical for tumor recurrence and patient survival (14). Patients with completely resected tumors have a significantly lower risk of tumor recurrence and metastasis and a higher 5-year survival rate compared to patients with incomplete tumor resection (15). Negative margins, tumor location, size, grade, and metastasis are also important factors affecting patient survival (14, 15). Complete surgical resection is more difficult for MPNSTs, considering the high degree of malignancy, early metastasis, and high mortality; therefore, adjuvant radiotherapy is important to improve survival, especially for patients with positive margins (14). However, the decision to administer adjuvant radiotherapy must be evaluated individually according to the patient’s medical status and risk of tumor recurrence (7). Radiotherapy is generally considered most effective for patients with larger tumors (> 5cm), high-grade tumors, and/or positive resection margins (3). Our team did not recommend routine postoperative adjuvant radiotherapy for this patient owing to a lack of definitive evidence that adjuvant radiotherapy for low-grade tumors results in time-to-survival gains. Additionally, a higher tumor grade is associated with a greater risk of poor prognosis (14). Anthracyclines are the first-line treatment option for patients with unresectable, advanced, or metastatic MPNSTs; furthermore, other cytotoxic chemotherapeutic agents (including the alkylating agent cyclophosphamide and the topoisomerase II inhibitor etoposide) may also be chosen (6). Adjuvant chemotherapy is considered important to improve the survival rate of patients with MPNSTs (14); however, the sensitivity of the tumor to chemotherapeutic agents needs to be clarified. The patient’s tumor grade was low owing to early diagnosis and treatment; therefore, we do not recommend conventional adjuvant radiotherapy (RT) and adjuvant chemotherapy (even with normal renal function) to avoid potential adverse effects.

Our patient had extremely poor renal function, requiring dialysis and other therapeutic means. The management of patients undergoing chemotherapy must be taken seriously owing to the potential myelosuppressive and hepatic and renal function impairments caused by most chemotherapeutic agents. Recent studies show a gradual increase in the use of adjuvant RT in patients with MPNSTs (16) with the application of novel RT techniques similar to Image Guided Radiation Therapy (IGRT), intensity modulation RT, volumetric modulated arc therapy (VMAT), stereotactic RT, and proton-based RT can effectively control tumor recurrence (16–18). However, the effect of adjuvant RT on patient survival is uncertain, making it a highly debated treatment option. Additionally, the use of DNA-damaging agents may lead to tumor recurrence or additional mutations, further complicating treatment decisions (3, 6, 7). A main challenge in the current advancement of medical technology is determining how to maximize the control of the radiation dose distribution and minimize the radiation to adjacent organs.

Nowadays, targeted therapy is an attractive treatment option for patients with metastatic or unresectable MPNSTs (3, 7). Cell signaling pathways and the tumor microenvironment are important areas for future research (19). Some studies suggest that the Ras/Raf and mTOR signaling pathways may be important in the pathogenesis of MPNSTs; and new target sites such as CDKN2A, Hsp90, BRAF V600E, and NRAS Q61 have been identified (6, 19). Although targeted therapeutic agents are less commonly used in clinical practice and have yielded inconsistent results, several animal-based experimental studies have shown positive outcomes (6, 19). Sex hormones may be associated with primary MPNSTs in the urinary tract (13); however, there are very few relevant cases, and more experimental studies or clinical cases are needed to confirm this. The investigation of targeted drugs for MPNSTs is a current research hotspot that could result in a significant advancement in treating patients with MPNSTs in the near future.

Choosing the appropriate treatment for patients with tumors with poor renal function is difficult. More clinical evidence is required to determine whether adjuvant chemotherapy based on renal function control is possible. We will also follow this patient for an extended period to obtain information regarding disease progression and treatment.

## Data availability statement

The original contributions presented in the study are included in the article/supplementary material. Further inquiries can be directed to the corresponding authors.

## Ethics statement

The studies involving humans were approved by Ethics Committee of The Guizhou Provincial People’s Hospital. The studies were conducted in accordance with the local legislation and institutional requirements. Written informed consent for participation was not required from the participants or the participants’ legal guardians/next of kin in accordance with the national legislation and institutional requirements. Written informed consent was obtained from the individual(s) for the publication of any potentially identifiable images or data included in this article. Written informed consent was obtained from the participant/patient(s) for the publication of this case report.

## Author contributions

XY: Resources, Writing – original draft, Writing – review & editing. XW: Methodology, Writing – review & editing, Writing – original draft. ZH: Resources, Writing – review & editing. XC: Investigation, Writing – review & editing. QW: Conceptualization, Validation, Writing – review & editing. KJ: Conceptualization, Validation, Writing – review & editing.
